# P05-12 Objectively measured physical activity, chronic illness and health service utilisation - a latent class analysis of activity behaviours in older adults

**DOI:** 10.1093/eurpub/ckac095.079

**Published:** 2022-08-29

**Authors:** Andrew O'Regan, Ailish Hannigan, Liam Glynn, Alan Donnelly, Grainne Hayes, Enrique Garcia Bengoechea, Catherine Woods

**Affiliations:** 1 School of Medicine, University of Limerick, Limerick, Ireland; 2 Health Research Institute and Department of Physical Education and Sports Science, University of Limerick, Limerick, Ireland

**Keywords:** older adults, physical activity, multi-morbidity, device-measured, latent class analysis

## Abstract

**Background:**

Physical activity contributes to the prevention of chronic illness as well as promotion of physical and mental health, but most adults remain inactive. Chronic illness affects mainly middle aged and older adults, and very little objectively measured data on physical activity behaviours and associated health outcomes of this population is published. The aims of this study are to: 1. Objectively measure physical behaviour outcomes of adults participating in the Move for Life study; 2. Develop distinct activity profiles based on six behaviour variables; 3. Investigate whether health outcomes differ across the activity profiles.

**Methods:**

Participants were Irish adults aged 50 years and older. Using the activPAL, objectively measured data were collected on average daily: light intensity physical activity (hours); moderate to vigorous intensity physical activity (minutes); step count; time in bed (hours); standing time (hours); and waking sedentary time (hours). Data were obtained on chronic illness and health service utilisation. Validated questionnaires were used to collect data on wellbeing, loneliness and social isolation. Hierarchical cluster analysis using squared Euclidian distance was used to cluster behaviours based on similarity, using SPSS version 26. Regression models explored associations between health outcomes and activity profiles, adjusted for age and sex.

**Results:**

Data from 485 participants were analysed, and four activity profiles were identified: sedentary (n = 50, 10.3% of total), low active (n = 295, 60.8%), moderate active (111, 22.9%) and higher active (n = 29, 6%). We will present the differences across the activity profiles for chronic illnesses, multi-morbidity, health service utilisation and validated health tools, comparing to data from the Irish Longitudinal Study on Ageing (TILDA) and the English Longitudinal Study on Ageing (ELSA).

**Conclusions:**

The use of physical activity behaviour clusters may identify people with multi-morbidity and higher utilisation of health services. These findings could be factored into the development of future targeted physical activity interventions.

